# The Macroautophagy Machinery in MHC Restricted Antigen Presentation

**DOI:** 10.3389/fimmu.2021.628429

**Published:** 2021-02-25

**Authors:** Christian Münz

**Affiliations:** Viral Immunobiology, Institute of Experimental Immunology, University of Zürich, Zürich, Switzerland

**Keywords:** cytotoxic CD8^+^ T cells, helper CD4^+^ T cells, cross-presentation, LC3-associated phagocytosis, exocytosis

## Abstract

Autophagy-related (ATG) gene products regulate macroautophagy, LC3-associated phagocytosis (LAP) and LC3-dependent extracellular vesicle loading and secretion (LDELS). These processes also influence antigen processing for presentation on major histocompatibility complex (MHC) molecules to T cells. Here, I summarize how these different pathways use the macroautophagy machinery, contribute to MHC class I and II restricted antigen presentation and influence autoimmunity, tumor immunology and immune control of infectious diseases. Targeting these different pathways should allow the regulation of intracellular and extracellular antigen presentation to T cells to modulate protective and pathological immune responses.

## The Macroautophagy Machinery

**Figure 1:**
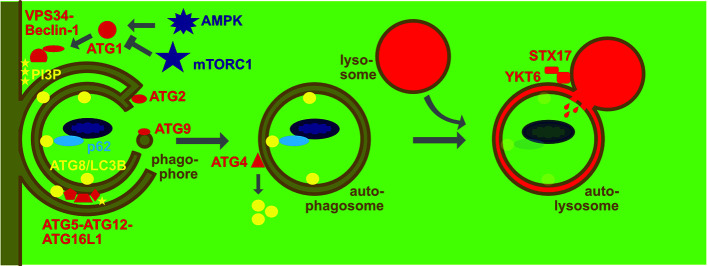
Molecular machinery of macroautophagy. The protein kinase ATG1/ULK1 is activated by AMP kinase (AMPK) and inhibited by mammalian target of rapamycin complex 1 (mTORC1). It then activates the lipid kinase complex containing VPS34 and Beclin-1 to generate phosphatidylinositol-3-phosphate (PI3P) that then recruits the ATG8/LC3B lipidation complex ATG5-ATG12-ATG16L1 that couples ATG8/LC3B to phosphatidylethanolamine at the inner and outer membrane of the phagophore. Membranes are donated to the phagophore *via* ATG2 associated channels and ATG9 carrying vesicles. Once this double membrane closes around cargo that is recruited by macroautophagy receptors like p62 *via* binding to ATG8/LC3 to form an autophagosome ATG4 recycles ATG8/LC3B from the outer membrane. The autophagosome fuses then with late endosomes and lysosomes in a syntaxin 17 (STX17) and YKT6 dependent fashion for degradation of the cargo and the inner autophagosome membrane with coupled ATG8/LC3B.

Yoshinori Ohsumi described in a landmark paper in 1993 15 genes that are required in yeast to survive starvation (1). These formed the core of the more than 40 autophagy-related proteins that regulate macroautophagy, one of at least three pathways by which cytoplasmic constituents are imported into lysosomes for degradation (2). This machinery consists of a protein kinase complex, a lipid kinase complex, enzymes that couple ubiquitin-like molecules to membranes and recruit substrates to them, as well as a fusion machinery that delivers the result of the first three complexes, a double-membrane surrounded autophagosome, to lysosomes for the degradation of the cargo and the inner membrane of autophagosomes (**Figure 1**). Many of the molecular components of this machinery are abbreviated as ATG (autophagy-related) proteins. The protein kinase complex of ATG1/ULK1 gets inhibited by mammalian target of rapamycin complex 1 (mTORC1) and activated by AMP kinase (AMPK) *via* differential phosphorylation. This allows eukaryotic cells to initiate macroautophagy upon nutrient deprivation. The ATG1/ULK1 complex then phosphorylates itself and components of all stages of autophagosome maturation and degradation (3). However, one of its main targets is the VPS34 phosphatidylinositol 3 (PI3) kinase complex containing ATG6/Beclin-1. This complex generates the phospholipid PI3P that recruits the ATG8/LC3B lipidation machinery to membranes *via* WIPI proteins, predominantly WIPI2. The ATG8/LC3B lipidation machinery consists of the E1-like enzyme ATG7. The E2-like enzymes ATG3 and 10, and the E3-like enzyme ATG5-ATG12-ATG16L1 that finally couples the six mammalian ATG8 orthologues LC3A, LC3B, LC3C, GABARAP, GABARAP-L1, and GABARAP-L2 primarily to phosphatidylethanolamine (PE) in the forming autophagosome that is called isolation membrane or phagophore. Attached to the phagophore membrane these ATG8 orthologues assist in membrane fusion during phagophore extension by ATG9 containing vesicles and ATG2 mediated lipid transfer (4–6). Prior to their lipidation the ATG8 orthologues need to be processed by the ATG4 proteases (ATG4A-D in higher eukaryotes) that also remove them from the outer membrane upon autophagosomes completion (7). The ATG8 orthologues also serve as anchors to recruit macroautophagy cargo to phagophores. Proteins with LC3-interacting regions (LIRs) bind to ATG8 orthologues and then the phagophore grows around the respective cargo, including damaged mitochondria, chloroplasts, ribosomes, proteasomes, endoplasmic reticulum, protein aggregates, damaged endosomes, bacteria, and some viral capsids. These LIR containing autophagy receptors, like sequestosome 1/p62 or NBR1 that cross-link for example ubiquitinated cytoplasmic constituents with LC3B (8). After removal of most lipidated ATG8 orthologues from the outer membrane of the completed autophagosome, residual autophagy receptor binding at this site supports autophagosome transport along microtubules and recruitment of the fusion machinery with lysosomes or late endosomes (9, 10). Finally, the soluble N-ethylmaleimide-sensitive-factor attachment receptors (SNAREs) syntaxin 17 and YKT6 are required for autophagosome fusion with lysosomes (11, 12). In the resulting autolysosome macroautophagy substrates and the inner autophagosomal membrane are degraded by lysosomal hydrolases. Therefore, macroautophagic flux is coupled to lysosomal activity and transcriptionally linked to transcription factor EB (TFEB) the master transcription factor of lysosomal biogenesis (13). The resulting molecular building blocks of lysosomal degradation, including amino acids, nucleic acids, sugars, and phospholipids are then recycled for energy generation and synthesis of new macromolecules to survive periods of starvation. Once such a catabolic machinery is in place it can be used for a multitude of other cellular processes, including degradation of pathogens, regulation of intercellular communication like inflammation and immune cell activation. In this review I will focus on its role in antigen presentation on major histocompatibility complex (MHC) molecules to T cells which utilizes proteolytic product display on MHC molecules at the cell surface.

## Macroautophagy in MHC Class II Restricted Antigen Presentation

The two classes of classical MHC molecules monitor different proteolytic machineries in cells by sampling a subset of these that they then transport to the cell surface for T cell stimulation (14–16). Pathogen-derived or otherwise foreign peptides can then be recognized by the T cell repertoire that has been tolerized against self-peptides. MHC class I molecules present primarily products of proteasomal degradation that are then imported *via* the transporter associated with antigen presentation (TAP) into the endoplasmic reticulum and loaded in the MHC-I peptide-loading complex (17). After transport to the cell surface MHC class I molecules and their presented mostly nonameric peptides are then screened by cytotoxic CD8^+^ T cells. In contrast MHC class II molecules are loaded with usually longer peptides but a nonameric core sequence in MHC class II containing compartments (MIICs) which are late endosomes with lysosomal proteolytic capacity. MHC class II molecules are transported to MIICs as complexes with the invariant chain (Ii) that is then degraded and peptides loaded with the assistance of HLA-DM (H2-M in mice) onto MHC class II molecules (18). MHC class II molecules with their bound peptide ligands then migrate to the cell surface for surveillance by helper CD4^+^ T cells.

**Figure 2:**
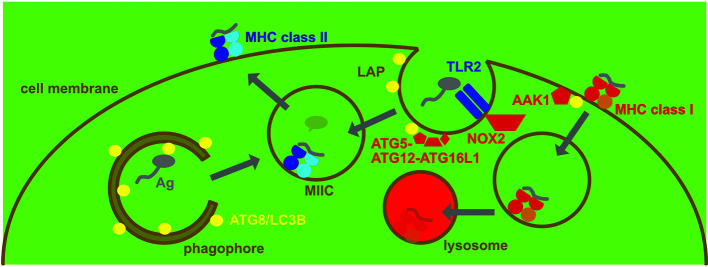
Regulation of MHC presentation by the macroautophagy machinery. MHC class II gets loaded with peptides in MHC class II containing vesicles (MIICs). They receive antigens (Ag) from intracellular sources *via* macroautophagy and from extracellular sources *via* phagocytosis, including LC3-associated phagocytosis (LAP). LAP recruits ATG8/LC3B to the phagosomal membrane after for example TLR2 engagement and is dependent on NADPH oxidase 2 (NOX2). The ATG8/LC3B lipidation complex of ATG5-ATG12-ATG16L1 conjugates ATG8/LC3B to the cytosolic side of phagosomes that then deliver the endocytosed antigen to MIICs. In contrast MHC class I restricted antigen presentation is restricted by the macroautophagy machinery, supporting MHC class I internalization and lysosomal degradation. Adaptor associated kinase 1 (AAK1) is recruited to MHC class I molecules for the respective internalization.

MHC class II restricted antigen presentation monitors therefore lysosomal proteolysis which degrades both endocytosed proteins and macroautophagy substrates (**Figure 2**). Indeed 20-30% of MHC class II ligands originate from cytosolic and nuclear sources, including the three ATG8 orthologues LC3B, GABARAP and GABARP-L2 (19, 20). MHC class II presentation of cytoplasmic constituents after macroautophagy was indeed initially demonstrated for three pathogen derived antigens, namely the nuclear antigen 1 of the Epstein Barr virus, bacterial neomycin phosphotransferase II (NeoR) and Ag85B of Mycobacterium tuberculosis (21–24). Processing of antigens for MHC class II presentation by macroautophagy can also be demonstrated by targeting them to phagophores *via* N-terminal conjugation to LC3B (25). This increases intracellular antigen presentation on MHC class II, but not MHC class I molecules. Furthermore, LC3B can be found in MIICs (25, 26). Enhanced presentation of antigens on MHC class II molecules after macroautophagy targeting has now been demonstrated for viral and tumor proteins (25, 27–30). The above listed studies demonstrate that macroautophagy contributes to antigen processing for MHC class II presentation in a variety of cell types, including B cells (19, 21, 22), epithelial cells (25), melanocytes (29) and myeloid antigen presenting cells (24, 28, 30). Therefore, MHC class II molecules can sample cytoplasmic antigens of pathogens for presentation *via* macroautophagy.

Possibly even more important, however, is the role of this pathway for CD4^+^ T cell tolerance induction. This tolerance induction requires in part MHC class II presentation by epithelial cells without significant phagocytic activity, such as thymic epithelial cells (TECs) (31). Medullary TECs (mTECs) express tissue-restricted self-antigens (TRAs) under the influence of the autoimmune regulator (AIRE). The intracellular expression of these TRAs leads to negative selection of autoimmune CD4^+^ T cell clones in the thymus to ensure tolerance of the T cell repertoire against self. It was shown that macroautophagy deficient thymi were not able to sufficiently perform this negative selection, resulting in colitis and multi-organ inflammation in mice (32). Moreover, this primarily affected antigens that are endogenously expressed at low levels and cannot be efficiently transferred to neighboring cells for uptake, as is the case for most TRA expression in mTECs (33). As for the pathogen derived antigens, targeting of these to phagophores *via* N-terminal conjugation to LC3B led to improved negative selection of CD4^+^ T cells. These findings suggest that efficient negative selection by TRAs in the thymus requires macroautophagy. Furthermore, regulatory CD4^+^ T cell stability in the periphery seems also to benefit from macroautophagy in dendritic cells (DCs) (34). However, this might not only depend on its role in endogenous self-antigen presentation on MHC class II molecules, but rather on a role of ATGs in co-stimulatory molecule expression. Nevertheless, macroautophagy can process cytoplasmic proteins for MHC class II presentation to CD4^+^ T cells and this seems particularly important for self-antigens that are expressed at low levels by cells with limited phagocytic potential in order to induce tolerance against these.

## LC3 Associated Phagocytosis in MHC Class II Restricted Antigen Presentation

In addition it was, however, already noted in the first study that abolished ATG5 expression in DCs to study the influence of the macroautophagy machinery on MHC restricted antigen presentation and T cell responses *in vivo* that also extracellular antigen presentation to CD4^+^ T cells benefits from ATGs (35). Indeed, apart from the ATG1/ULK1 protein kinase complex the other components of the macroautophagy machinery also regulate phagocytosis. This was coined LC3-associated phagocytosis (LAP) (**Figure 2**), and the respective LC3B conjugation to the cytosolic side of phagosomes is primarily observed after co-engagement of pathogen associated molecular pattern receptors, such as toll-like receptor (TLR) 2, during uptake of extracellular material (36–40). During LAP, PI3P is deposited in a VPS34 dependent manner at the cytosolic side of phagosomes (41). This might be required to assemble efficiently NADPH oxidase (NOX2) at these phagosomes (42) and NOX2 mediated reactive oxygen species (ROS) production is required for LAP (37, 41, 43). The exact role of this ROS production is unclear to date, but the recruitment of the ATG8/LC3B lipidation seems to neither depend on ROS not PI3P at phagosomes (44). Instead it depends on the WD40 domain of ATG16L1 that is not required for macroautophagy (44, 45). The ATG16L1 dependent conjugation of LC3B to the cytosolic side of phagosomes is then removed prior to fusion with lysosomes as was observed by live cell imaging (36, 37). Dependent on the cell type LAP leads to accelerated fusion with lysosomes, delayed phagosome maturation or redirection of phagocytosed cargo to TLR containing endosomes (36, 37, 46). This probably depends on the recruitment of different vesicular transport factors to LAP phagosomes in different cellular backgrounds.

Nevertheless, in both human and mouse phagocytes LAP supports MHC class II presentation of endocytosed antigens (37, 39, 44, 47, 48). Since yeast cell wall components efficiently engage TLR2 Zymosan and Candida albicans spores or extracts were often used in these assays, and MHC class II presentation to Candida specific Th17 cells was monitored (37, 39). Phagocytosed Candida antigen presentation on MHC class II molecules to Th17 cells requires ATG5, ATG16L1 and more specifically the WD40 domain of ATG16L1 (37, 39, 44). Bacterial outer membrane vesicles (OMVs) might also be processed *via* this LAP for regulatory CD4^+^ T cell stimulation (49). In addition to TLR mediated LAP induction it was also described that B cell stimulation activates ATG1/ULK1 independent use of the macroautophagy machinery (50). Accordingly, B cell receptor (BCR) mediated antigen uptake was found to use the ATG8/LC3B lipidation machinery (51, 52). This allows BCR bound antigens to be delivered to TLR containing endosomes and to MIICs for antigen processing towards MHC class II presentation. Finally, myelin autoantigen presentation by DCs in the central nervous system (CNS) also depends on ATG5 and NOX2 as hallmarks of LAP, even so the receptor that mediates LC3B recruitment to phagocytosed oligodendrocyte derived apoptotic blebs has not been identified yet (47, 48). However, in mouse macrophages the phosphatidylserine binding scavenger receptor TIM4 was found to be involved in the clearance of apoptotic bodies by LAP (38). Thus, LAP supports autoimmune CD4^+^ T cell stimulation in the CNS for experimental autoimmune encephalomyelitis (EAE) development.

Therefore, ATG proteins support both intracellular and extracellular antigen presentation on MHC class II molecules to CD4^+^ T cells *via* macroautophagy and LAP, respectively.

## Regulation of MHC Class I Restricted Antigen Presentation by the Macroautophagy Machinery

In contrast to the support of MHC class II restricted antigen presentation by the macroautophagy machinery, loss of ATGs leads to up-regulation of MHC class I restricted presentation of intracellular antigens (53–57). This affects both classical MHC class I molecules and the non-classical MHC class I molecule CD1D that restricts glycolipid presentation to NKT cells. In these studies it was found that the stimulation of anti-viral, anti-tumor and alloreactive CD8^+^ T cell responses as well as anti-bacterial NKT cell immunity is enhanced in the absence of ATG3, ATG5, ATG7 or ATG16L1 (53–56). Higher classical and non-classical MHC class I expression on the surface of DCs and pancreatic carcinoma cells is at least in part responsible for this increased stimulation (54–56). This elevated surface expression of MHC class I molecules seems to be due to MHC class I targeting for lysosomal degradation by ATG proteins (**Figure 2**). In DCs this seems to be due to increased internalization and then degradation with the support of the macroautophagy machinery (54, 56). The identification of the adaptor associated kinase 1 (AAK1) and the adaptor complex AP2 point towards ATG proteins supporting clathrin mediated endocytosis (54, 56). This is reminiscent of the internalization of the amyloid precursor protein (APP) in Alzheimer’s disease that was reported to require LC3 mediated recruitment of AP2 for efficient clathrin mediated turn-over (58, 59). In contrast in pancreatic carcinoma cells an NBR1 dependency of MHC class I degradation was observed and the authors suggested that macroautophagy of ER might redirect MHC class I molecules to lysosomes and therefore diminish surface expression for CD8^+^ T cell stimulation (55). In both instances, deficiencies in the macroautophagy machinery, however, increase anti-viral (influenza and lymphocytic choriomeningitis virus) and anti-tumor CD8^+^ T cell responses *via* increased surface expression of MHC class I molecules. Lysosomal degradation of MHC class I molecules by the macroautophagy machinery seems to be also induced by ORF8 of the severe acute respiratory syndrome coronavirus 2 (SARS-CoV-2) (60). Moreover, autophagy inhibition can also redirect intracellular antigen degradation to proteasomes leading to increased MHC class I antigen presentation (57). Therefore, multiple pathways might account for increased MHC class I presentation of intracellular antigens by both somatic cells, such as tumor cells, and antigen presenting cells upon inhibition of the macroautophagy machinery.

With respect to extracellular antigen processing for MHC class I presentation, so called cross-presentation, the contribution of the macroautophagy machinery is not entirely clear yet. So far only soluble proteins have been described to benefit from the macroautophagy machinery in cross-presenting classical type 1 DCs and B cells (61, 62). However, long-term storage of antigen by DCs for cross-presentation was rather compromised by the macroautophagy machinery (63). Another function for antigen cross-presentation on MHC class I molecules was described for the macroautophagy machinery in antigen donor cells like virus-infected stromal or tumor cells (64, 65). In these studies, exocytosis of antigen containing vesicles that get efficiently cross-presented for CD8^+^ T cell stimulation by DCs seemed to benefit from the macroautophagy machinery. The packaging of antigens into the respective vesicles was improved by inhibiting both proteasomal and lysosomal degradation in the antigen donor cells (66–70). The respective pathway that utilizes the ATG8/LC3B lipidation machinery for cross-presentable vesicle export could be overlapping or identical to LC3-dependent extracellular vesicle loading and secretion (LDELS) (71–76). Potentially more than one exocytosis pathway seems to benefit from the macroautophagy machinery and neutral sphingomyelinase 2 (nSMase2) or Golgi reassembly stacking proteins (GRASPs) have been reported to be involved in this ATG supported exocytosis. Thus, extracellular antigen processing for MHC class I presentation seems to benefit from a functional macroautophagy machinery in both antigen donor cells and for short-term cross-presentation of certain antigen formulations also in antigen presenting cells, while the autophagic machinery limits MHC class I surface expression for intracellular antigen presentation.

## Conclusions and Future Perspectives

Lysosomal and proteasomal protein degradation are the two main proteolytic machineries of cells and their products sampled by MHC class II and I molecules for CD4^+^ and CD8^+^ T cell stimulation, respectively. Accordingly, macroautophagy as a component of lysosomal degradation, targeting cytoplasmic constituents, supports MHC class II restricted antigen presentation to helper CD4^+^ T cells. Recent years have, however, demonstrated that the same molecular machinery that supports macroautophagy also regulates endocytosis and exocytosis. While the role of ATG proteins in endocytosis seems to promote MHC class II presentation of phagocytosed antigens *via* LAP and inhibit MHC class I presentation of intracellular antigens through lysosomal degradation of MHC class I plus peptide complexes, exocytosis might package antigens optimally for processing onto MHC class I molecules during cross-presentation. Since not all modules of the macroautophagy machinery are used in all these pathways, regulation of specific ATG proteins in antigen donor or presenting cells might be used to influence MHC class I or II presentation in the tumor microenvironment or during viral infections. However, which manipulation might be beneficial in which setting needs to be determined on a case by case basis, considering, in addition to the role of the macroautophagy machinery for MHC restricted antigen presentation, also its anti-inflammatory role in myeloid cells and its pro-survival role in lymphocytes as well as in virus infected and tumor cells.

## Author Contributions

The author confirms being the sole contributor of this work and has approved it for publication.

## Funding

My research is supported by Cancer Research Switzerland (KFS-4091-02-2017 and KFS-4962-02-2020), KFSP-Precision^MS^ and HMZ ImmunoTargET of the University of Zurich, the Cancer Research Center Zurich, the Vontobel Foundation, the Baugarten Foundation, the Sobek Foundation, the Swiss Vaccine Research Institute, Roche, Novartis and the Swiss National Science Foundation (310030B_182827, 310030L_197952/1, and CRSII5_180323).

## Conflict of Interest

The author declares that the research was conducted in the absence of any commercial or financial relationships that could be construed as a potential conflict of interest.
